# Enhancing Soybean Yield: The Synergy of Sulfur and Rhizobia Inoculation

**DOI:** 10.3390/plants12223911

**Published:** 2023-11-20

**Authors:** Yiao Hu, Yulin Chen, Xu Yang, Lansheng Deng, Xing Lu

**Affiliations:** 1 College of Natural Resources and Environment, South China Agricultural University, Guangzhou 510642, China; huyiao1991@163.com (Y.H.); cyl0912@stu.scau.edu.cn (Y.C.); jeneryang819@163.com (X.Y.); xinglu@scau.edu.cn (X.L.); 2 Guangdong Weisheng Liansu Technology Co., Ltd., Foshan 528313, China

**Keywords:** soybean, nitrogen fixation, sulfur fertilization, rhizobia inoculation, root nodules, root morphology

## Abstract

Sulfur deficiency severely limits soybean growth, inhibiting the rhizobia nitrogenase and soybean protein synthesis. This study assessed the impact of sulfur fertilization and rhizobia inoculation on soybean growth and nitrogen fixation through bacterial culture and hydroponic experiments. We selected three rhizobia strains for bacterial cultures and used six sulfur levels. The test demonstrated severe inhibition of *Rhizobium* USDA110 growth without sulfur. In hydroponic experiment, we employed five sulfur levels with USDA110 as the inoculum strain. Soybean growth, nitrogen fixation, yield, and root morphology-related parameters, and root nodule growth, were significantly inhibited without sulfur. Following *Rhizobium* inoculation, low sulfur concentrations (0.15–0.60 mM) stimulated early-stage (V9) root growth and increased shoot nitrogen accumulation, but inhibited root growth at R5 stage. Furthermore, *Rhizobium* inoculation notably enhanced soybean growth, nitrogen fixation, and yield, especially within the recommended low sulfur concentration range (0.15–0.30 mM). The maximum nodule nitrogenase activity at R5 stage and highest yield was recorded at a 0.3 mM sulfur concentration with *Rhizobium* inoculation, which was 9.51–1222.07% higher than other treatments. These findings highlight that low sulfur concentration and rhizobia inoculation enhance soybean growth, nitrogen fixation, and yield but reduce soybean root efficacy, increasing reliance on root nodules.

## 1. Introduction

Soybean (*Glycine max* (L.) Merr.), is a crop belonging to the legume family [1]. Globally, soybean occupies a significant position both as an oil crop and a protein-rich resource [2]. Notably rich in high-quality protein, fats, dietary fiber, vitamins, and minerals, soybeans offer substantial nutritional value [3]. Consequently, it is widely recognized as a vital plant-based protein source with far-reaching implications for human and animal health as well as nutrition. The contemporary surge in health-conscious dietary preferences, alongside the rise of vegetarianism, has underscored an increasing consumer demand for plant-based protein sources [4]. This trend has particularly cast a spotlight on soybean, celebrated for its abundance of high-quality protein [5]. Based on data sourced from the Food and Agriculture Organization of the United Nations (FAO), the global cultivation area for soybeans has exhibited a consistent annual increase since the year 2000 [6]. Notably, by the year 2021, the worldwide soybean cultivation area had expanded significantly to encompass 129,523,964 hectares—an impressive uptick of 74.31% in comparison to the figures recorded in 2000 [6]. In contrast, China’s soybean yield per unit area in 2021 showed a mere 10.74% increase compared to the average yield of the preceding 21 years (2000–2020) [6]. With the steady growth of the global population and ongoing economic development, the demand for soybeans continues to rise persistently. In light of these circumstances, enhancing the yield per unit area of soybean cultivation emerges as a matter of paramount significance in upholding worldwide food security.

Nitrogen sources abound in nature, with approximately 80% of the atmosphere consisting of nitrogen. Within the framework of nitrogen fixation, nitrogen-fixing bacteria play a pivotal role, accounting for 60% of the fixed nitrogen [7]. Among these processes, the direct symbiotic nitrogen fixation between legumes and rhizobia stands out as a cornerstone in biological nitrogen fixation [8]. Plants that host root nodules can be categorized into two primary types: indeterminate and determinate [9]. Soybean belongs to the determinate type of root nodules [10].

*Rhizobium* bacteria effectively infiltrate the root cells of legumes, rapidly proliferating within them and giving rise to bacteroides [11]. Upon the emergence of soybean nitrogenase in the root nodules, atmospheric nitrogen undergoes reduction to ammonia [12]. This ammonia subsequently reacts with water to form ammonium ions, which are available for absorption and utilization by plants [13]. In root nodules, legume hosts assimilate ammonium ions, converting them into nitrogen-rich organic compounds like amino acids and proteins [14]. These compounds contribute to the plant’s growth and developmental processes.

Upon the inoculation of soybeans with rhizobia, a notable augmentation in the rhizobia population occurs [15]. This increase parallels the heightened activities of both rhizobia and soil microorganisms [16]. Consequently, the capacity for nitrogen fixation experiences significant enhancement [17]. Harnessing the symbiotic nitrogen fixation between leguminous crops and rhizobia offers multifaceted benefits. It not only ameliorates soil fertility and fulfills nitrogen requirements of crops but also curtails the reliance on chemical nitrogen fertilizers, thereby mitigating the environmental pollution stemming from excessive nitrogen fertilizer application [18].

Sulfur (S) stands as a fundamental mineral nutrient imperative for comprehensive plant growth and development. Its engagement is principally witnessed in pivotal physiological and biochemical mechanisms, encompassing photosynthesis, respiration, nitrogen fixation, protein, and lipid synthesis, all of which profoundly influence plant maturation [19]. Within proteins, sulfur emerges as an elemental constituent, pivotal for orchestrating protein synthesis and indispensably contributing to protein folding and structural stability [20]. Furthermore, sulfur induces alterations in the chemical configuration of oleic acid in plants, thereby augmenting the fatty acid reservoir and subsequently elevating oil production [21]. It is noteworthy that distinct plant species manifest varying prerequisites for sulfur supplementation. Soybean crops are both legumes and oil crops, requiring more sulfur to support protein and oil synthesis processes [22].

While the overall sulfur content in soil remains modest, most crops typically demonstrate resilience against sulfur deficiency [23]. Research has elucidated that introducing sulfur to deficient soil yields the dual benefits of bolstering nitrogen fixation and enhancing soybean yield and quality [24]. Sulfur assumes a multifaceted role encompassing protein synthesis, metabolic processes, electron transfer, urease, and coenzyme A functions [25,26]. This dynamic engagement extends further to implicate soybean nitrogen metabolism, chlorophyll content, protein, and fat levels [27]. Notably, ferredoxin emerges as a pivotal sulfur-containing compound. It operates not only in the reduction of carbon dioxide during the dark phase of photosynthesis but also contributes to sulfate reduction, nitrogen reduction, and glutamic acid synthesis [28]. Sulfur is underscored by its role as a critical constituent within the Fe protein and Fe-Mo protein components of the nitrogenase complex situated in legume root nodules. Beyond its role in symbiotic nitrogen fixation, sulfur exerts influence over plant root growth dynamics [29].

In regions of southern China, characterized by acidic pH levels, soil sulfur predominantly exists in sulfate form [30]. However, owing to its tropical and subtropical location, enhanced precipitation contributes to heightened sulfate solubilization in the soil, with subsequent migration to water bodies. This phenomenon escalates the risk of soil sulfur deficiency. Reports indicate a mounting prevalence of soil sulfur deficiency in the agricultural domains of the country over the past decades. Gradually, this deficiency has evolved into a limiting factor for crop yields in various global regions [31,32]. Considering the significant demand for sulfur in soybeans and the soil sulfur deficiency, the application of sulfur-based fertilizers remains a pivotal strategy to uphold soybean yield stability. Consequently, to unravel the influence of sulfur on soybean root nodule growth and nitrogen fixation, this study embarked on the identification of low-sulfur-sensitive rhizobia via bacterial culture experiment. Moreover, a hydroponic experiment was conducted to explore whether the stimulation of sulfur supply impacting host plant growth stemmed from the following factors: (i) sulfur-induced rhizobia stimulation, (ii) rhizobia-root interaction effects, and (iii) nodulation-induced nutrient uptake effects.

## 2. Results

### 2.1. Screening of Rhizobia

**Figure 1:**
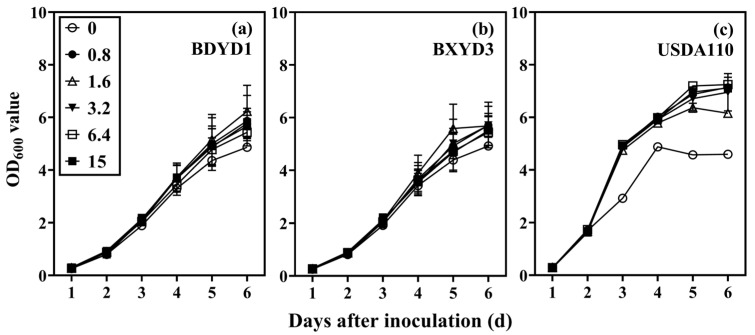
The reproduction of BDYD1 rhizobia (**a**), BXYD3 rhizobia (**b**), and USDA110 rhizobia (**c**) was studied under various sulfur concentrations. The data presented in the figure represent the average of four replicates. The values shown in the figure represent the optical density (OD) measurements taken after continuously culturing each strain for 6 days.

The growth of the three types of rhizobia changed rapidly after 6-day culture in a YMB liquid medium (Figure 1). For BDYD1 and BXYD3, there was no significant difference in their growth response to different sulfur concentrations (BDYD1: *F* = 0.037, *p* = 0.999; BXYD3: *F* = 0.028, *p* = 0.999; Figure 1a,b). However, the growth of USDA110 was notably inhibited at 0 mM sulfur concentration, exhibiting a significant difference compared to other sulfur concentrations (*p* < 0.01; Figure 1c). On days 3 to 7, the OD value at 0 mM sulfur concentration decreased by 40.36%, 19.09%, 33.49%, and 35.68%, respectively, compared to the 0.8 mM sulfur concentration. This indicates that low sulfur concentration significantly impacted the growth of USDA110, but it did not have a significant effect on BDYD1 and BXYD3. Therefore, USDA110 rhizobium bacteria, which are sensitive to low sulfur concentration, were selected as the test material for this experiment.

### 2.2. Root Lengths and Specific Root Lengths

**Figure 2:**
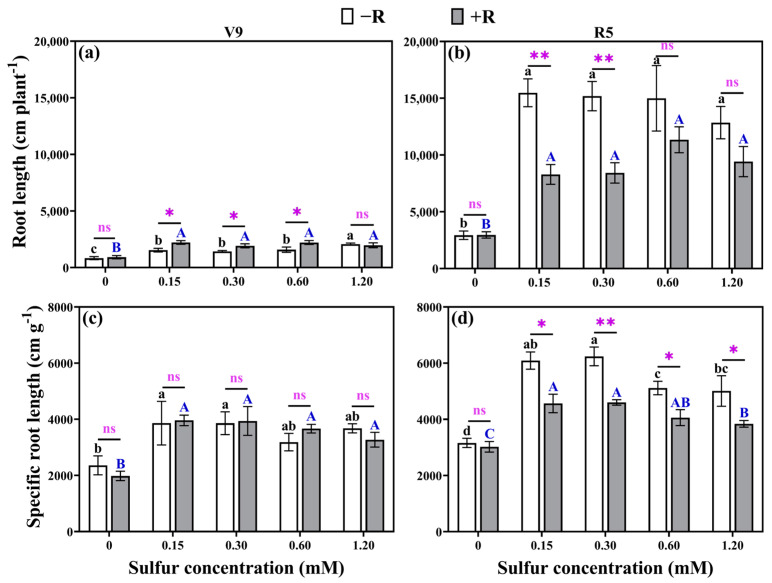
Root lengths at the V9 stage (**a**) and R5 stage (**b**), as well as specific root lengths at the V9 stage (**c**) and R5 stage (**d**), were measured for both uninoculated rhizobium and rhizobium-inoculated treatments under various sulfur concentrations. (−R) no rhizobia inoculation and (+R) rhizobia inoculation. The same lowercase letters indicate that the different sulfur concentration treatments without rhizobia inoculation showed no significant differences (*p* > 0.05). The same capital letters indicate that the different sulfur concentration treatments with rhizobia inoculation showed no significant differences (*p* > 0.05). The asterisks (*, **) denote a significant difference between uninoculated and inoculated rhizobia at the same sulfur concentration; * indicates *p* < 0.05, ** indicates *p* < 0.01, and “ns” indicates no significant difference (*p* > 0.05) between uninoculated and inoculated rhizobia at the same sulfur concentration. Root length (RL) refers to the total length of all the roots of the plant. Specific root length (SRL) refers to the ratio of root length to root dry weight.

Significant differences were observed in root length and specific root length of soybean among different sulfur concentrations and rhizobia inoculation methods (Figure 2). Irrespective of rhizobial inoculation, the inclusion of sulfur resulted in a notable increase in total root length, ranging from 72.16% to 427.86%, and specific root length, spanning 26.96% to 99.76%, in comparison to the 0 mM sulfur concentration at both the V9 and R5 stages, providing strong evidence that the application of sulfur promotes soybean root growth. However, the root length did not show a significant increase with higher sulfur concentrations, except for the uninoculated rhizobia treatment at a sulfur concentration of 1.20 mM (Figure 2a,b). This suggested that there was no direct positive correlation between sulfur concentration and soybean root growth. Regarding specific root length, it exhibited an increasing trend with lower sulfur concentrations and a decreasing trend with higher sulfur concentrations (Figure 2c,d), indicating that lower sulfur concentrations promoted the growth of fine roots, while higher sulfur concentrations inhibited their growth. With the exception of the 1.20 mM sulfur concentration, a notable surge in root length, ranging from 34.01% to 43.95%, was evident at the V9 stage in rhizobia-inoculated treatments when compared to their non-inoculated counterparts within the sulfur-supplemented group. However, the specific root length of the treatment inoculated with rhizobia did not increase (Figure 2a,c). This result indicated that rhizobia inoculation promoted the overall soybean root growth during the early growth stage but limitedly increased the proportion of fine roots. In contrast, at the R5 stage within the sulfur-supplemented group, treatments lacking rhizobia demonstrated a notably higher root length and specific root length compared to their rhizobia-inoculated counterparts, with increments ranging from 32.23% to 86.71% and 25.98% to 35.59%, respectively (Figure 2b,d). This indicated that rhizobia inoculation reduced the growth of fine roots and the proportion of fine roots in the later stage of soybean growth.

### 2.3. Number and Dry Weight of Nodules

Significant variations were observed in the number and dry weight of soybean nodules among different sulfur concentrations and growth stages (Table 1). The number of small and medium-sized nodules surpassed that of larger nodules. Furthermore, the quantity of nodules with a diameter of 0–3 mm displayed an upward trend with increasing sulfur concentration at the V9 stage, surging by 333.33% at the 1.20 mM sulfur concentration compared to the 0.15 mM sulfur concentration. However, at the R5 stage, this trend reversed, leading to a 201.33% decrease in the number of 0–3 mm nodules at the 1.20 mM sulfur concentration compared to the 0.30 mM sulfur concentration. Conversely, the number of nodules with a diameter greater than 3 mm did not show significant changes with increasing sulfur concentration at both the V9 and R5 stages.

At the V9 stage, there was a rising trend in the number of 0–3 mm nodules as sulfur concentration increased. The 1.20 mM sulfur concentration witnessed a remarkable 333.33% increase compared to the 0.15 mM sulfur concentration. Additionally, both 0–3 mm nodules and larger nodules exhibited an increase in dry weight at this stage. Specifically, the 1.20 mM sulfur concentration showed a 159.68% increase in dry weight for 0–3 mm nodules and a 185.71% increase for larger nodules in comparison to the 0.15 mM sulfur concentration. However, at the R5 stage, while higher sulfur concentrations led to an increase in the dry weight of larger nodules, the dry weight of 0–3 mm nodules decreased by 57.03% at the 1.20 mM sulfur concentration compared to the 0.15 mM sulfur concentration. As a result, the trend reversed at this stage, with only the larger nodules displaying an increase in dry weight. Specifically, the 1.20 mM sulfur concentration exhibited a 201.09% increase in dry weight compared to the 0.15 mM sulfur concentration.

### 2.4. Nitrogenase Activity

**Figure 3:**
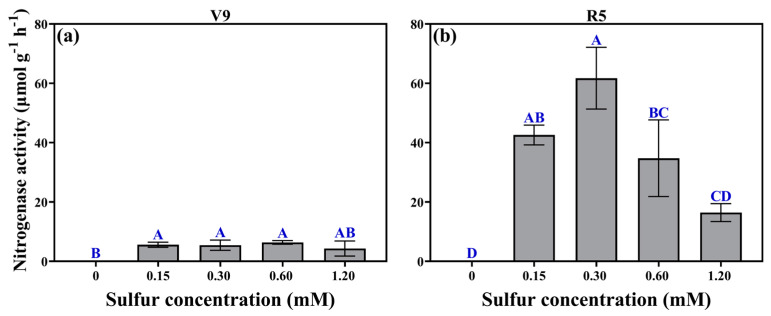
At the V9 stage (**a**) and R5 stage (**b**), after rhizobial inoculation, nitrogenase activity was measured for different sulfur concentration treatments. The same capital letters indicate that the different sulfur concentration treatments inoculated with rhizobia did not differ significantly (*p* > 0.05).

No statistically significant difference was shown in nitrogenase activity among nodules with different sulfur concentrations (*F* = 2.358, *p* = 0.112) at the V9 stage (Figure 3a). However, at the R5 stage, except for the 0 mM sulfur concentration, nitrogenase activity significantly increased across all sulfur concentrations compared to the V9 stage (*F* = 6.898, *p* = 0.008), indicating substantial variations in nitrogenase activity among nodules with different sulfur concentrations. Remarkably, the highest nitrogenase activity recorded was 61.72 μmol g^−1^ h^−1^ at a sulfur concentration of 0.3 mM, representing a substantial increase of 275.65% compared to the 1.20 mM sulfur concentration. Furthermore, a notable difference in nitrogenase activity was observed between nodules with different sulfur concentrations, underscoring the profound impact of sulfur concentration on nitrogenase activity during the R5 stage. These results provide robust support for the hypothesis that sulfur availability plays a pivotal role in nitrogen fixation by influencing the activity of nitrogenase enzymes.

### 2.5. Nodule Nitrogen Concentration and Accumulation

**Figure 4:**
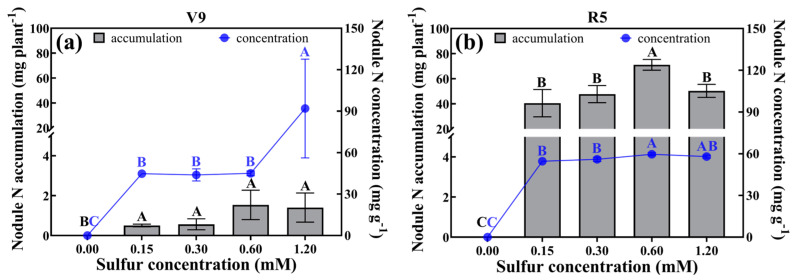
At the V9 stage (**a**) and R5 stage (**b**), after rhizobial inoculation, nodule nitrogen accumulation and nodule nitrogen concentration under various sulfur concentration treatments were examined. If different sulfur concentration treatments inoculated with rhizobia were not significantly different (*p* > 0.05), they are denoted by identical capital letters of the same color.

As illustrated in Figure 4, there was a noteworthy disparity in nodule nitrogen concentration and accumulation between the treatments with and without sulfur addition. At the V9 stage, the nodule nitrogen concentration exhibited a substantial increase of 104.24% to 109.60% in the treatment with a sulfur concentration of 1.20 mM, in comparison to other sulfur concentration treatments. Nonetheless, no significant differences were observed in the nitrogen accumulation within the nodules among the various sulfur addition treatments. Transitioning to the R5 stage, both nodule nitrogen concentration and accumulation were higher by 2.88–9.16% and 41.24–75.52%, respectively, in the treatment with a sulfur level of 0.60 mM, relative to the other treatments.

### 2.6. Nitrogen Accumulation

**Figure 5:**
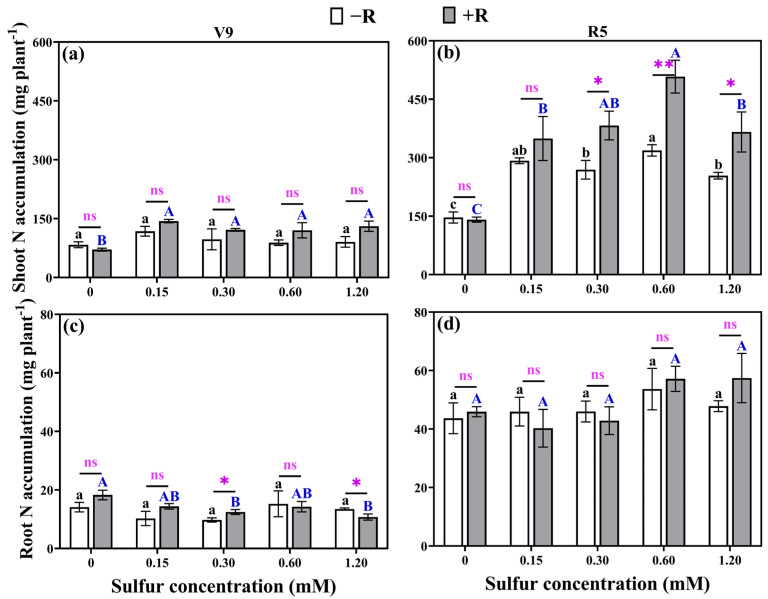
The shoot nitrogen accumulation at the V9 stage (**a**) and R5 stage (**b**), as well as the root nitrogen accumulation at the V9 stage (**c**) and R5 stage (**d**), were measured for both uninoculated rhizobium and rhizobium-inoculated treatments under various sulfur concentrations. (−R) no rhizobia inoculation and (+R) rhizobia inoculation. The same lowercase letters indicate that the different sulfur concentration treatments without rhizobia inoculation showed no significant differences (*p* > 0.05). The same capital letters indicate that the different sulfur concentration treatments with rhizobia inoculation showed no significant differences (*p* > 0.05). The asterisks (*, **) denote a significant difference between uninoculated and inoculated rhizobia at the same sulfur concentration; * indicates *p* < 0.05, ** indicates *p* < 0.01, and “ns” indicates no significant difference (*p* > 0.05) between uninoculated and inoculated rhizobia at the same sulfur concentration.

At the V9 stage, the shoot nitrogen accumulation with rhizobial inoculation was slightly higher than that of the non-inoculated treatment but without statistical significance. However, the combination of sulfur addition and rhizobial inoculation led to a substantial increase in aboveground nitrogen accumulation by 63.19–134.65% when compared to treatments that included rhizobial inoculation but lacked added sulfur (Figure 5a). During the V9 stage, root nitrogen accumulation was significantly reduced by 31.75% and 41.40% in treatments inoculated with rhizobia at sulfur concentrations of 0.30 mM and 1.20 mM, respectively, in contrast to the treatment with rhizobia but without added sulfur (Figure 5c). At later growth stages (R5), there were no significant differences in nitrogen content among the different root groups, indicating that sulfur concentrations and rhizobial inoculation had no significant effect on nitrogen accumulation in soybean roots. However, aboveground nitrogen accumulation exhibited a significant reduction of 42.25–72.23% in treatments without added sulfur when compared to treatments with added sulfur. Rhizobial inoculation in combination with added sulfur significantly increased aboveground nitrogen accumulation (Figure 5b). The highest aboveground nitrogen accumulation was observed in the treatment inoculated with *Rhizobium* and featuring a sulfur concentration of 0.60 mM, representing a substantial increase of 260.12% compared to the treatment inoculated with *Rhizobium* but lacking sulfur supplementation (Figure 5b). These findings underscore the capacity of sulfur addition and rhizobial inoculation to enhance aboveground nitrogen accumulation in soybean.

### 2.7. Dry Weight and Yield

**Figure 6:**
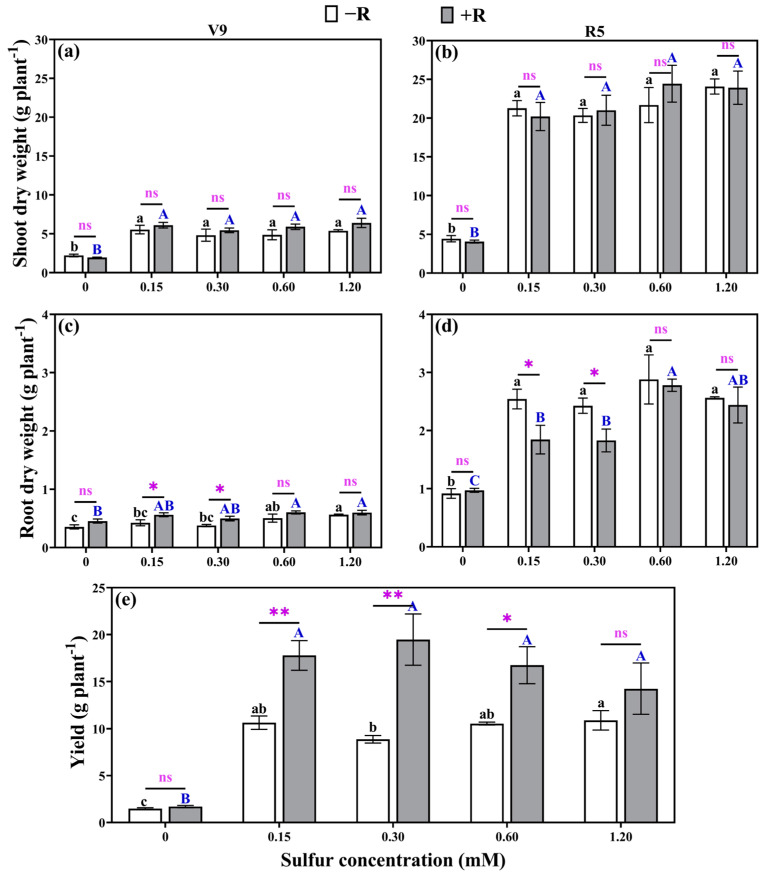
The shoot dry weight at the V9 stage (**a**) and R5 stage (**b**), root dry weight at the V9 stage (**c**) and R5 stage (**d**), and yield (**e**) were measured for both uninoculated rhizobium and rhizobium-inoculated treatments under various sulfur concentrations. (−R) no rhizobia inoculation and (+R) rhizobia inoculation. The same lowercase letters indicate that the different sulfur concentration treatments without rhizobia inoculation did not show significant differences (*p* > 0.05). The same capital letters indicate that the different sulfur concentration treatments with rhizobia inoculation did not show significant differences (*p* > 0.05). Asterisks (*, **) denote a significant difference between uninoculated and inoculated rhizobia at the same sulfur concentration; * indicates *p* < 0.05, ** indicates *p* < 0.01, and “ns” indicates no significant difference (*p* > 0.05) between uninoculated and inoculated rhizobia at the same sulfur concentration.

Figure 6 illustrated the variations in shoot and root dry weight, as well as grain yield of soybean under different sulfur concentrations and rhizobial inoculation. During both the V9 and R5 stages, rhizobia inoculation failed to increase shoot dry weight. Nonetheless, in the presence of added sulfur, irrespective of concentration and rhizobial inoculation, aboveground dry weight exhibited a significant increase. Specifically, at the V9 stage, soybeans displayed a notable boost in dry weight, ranging from 117.99% to 230.03%, while at the R5 stage, the increase was even more substantial, varying from 355.18% to 500.49%, when compared to soybeans without sulfur supplementation (Figure 6a,b). Lower sulfur concentrations (0.15 mM and 0.30 mM) led to an increase in soybean root dry weight at the V9 stage but resulted in reduced root dry weight at the R5 stage, especially when rhizobium inoculation was present (Figure 6c,d). Overall, soybean yields inoculated with rhizobium were significantly higher than those without inoculation (Figure 6e). Soybean yield was severely affected when no sulfur was added (0 mM), resulting in a significantly lower yield compared to sulfur addition. In the absence of rhizobia (−R) inoculation, soybean yield without sulfur addition showed reductions of 86.16%, 83.40%, 86.03%, and 86.48%, respectively, compared to soybean with sulfur addition. Under rhizobia (+R) inoculation, soybean yield without sulfur addition exhibited reductions of 90.48%, 91.31%, 89.89%, and 87.10%, respectively, in comparison to soybean with sulfur addition. A sulfur concentration of 0.3 mM, combined with *Rhizobium* inoculation, resulted in the highest yield at 19.47 g per plant, surpassing the yields of other treatments by a notable range of 9.51–1222.07%. However, when the sulfur concentration exceeded 0.15 mM, the yield did not significantly increase further. Therefore, considering cost-effectiveness, soybean planting should include sulfur supplementation, with the optimum condition being 0.15–0.30 mM.

### 2.8. Principal Component Analysis (PCA)

**Figure 7:**
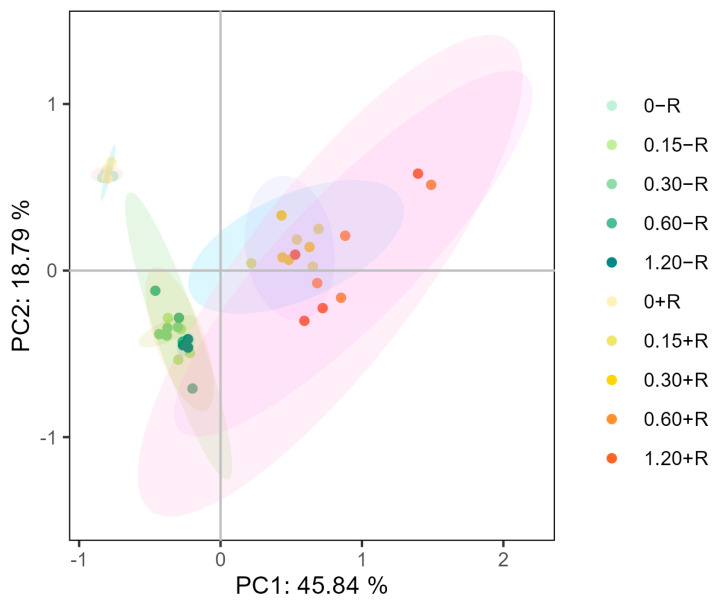
Principal component analysis (PCA) was conducted on soybean plant samples under different sulfur concentrations, comparing rhizobia in uninoculated and inoculated conditions.

By integrating all the aforementioned data, a principal component analysis (PCA) was conducted to investigate potential differences in soybean growth and yield concerning various sulfur concentrations and rhizobia inoculation. As depicted in Figure 7, when the sulfur concentration was 0 mM, the two treatments were closely grouped, regardless of rhizobia inoculation. However, the sample points with sulfur addition but no rhizobia inoculation, as well as those with sulfur addition and rhizobia inoculation, showed good dispersion and were not clustered together. This suggested that rhizobia inoculation was necessary to impact the growth and yield of soybeans under sulfur addition conditions. Nonetheless, regardless of rhizobia inoculation (+R) or non-inoculation with rhizobia (−R), there was minimal dispersion of sample points with increasing sulfur concentration, indicating that sulfur concentration had little effect on soybean growth and yield.

### 2.9. Pearson Correlation Coefficients

**Figure 8:**
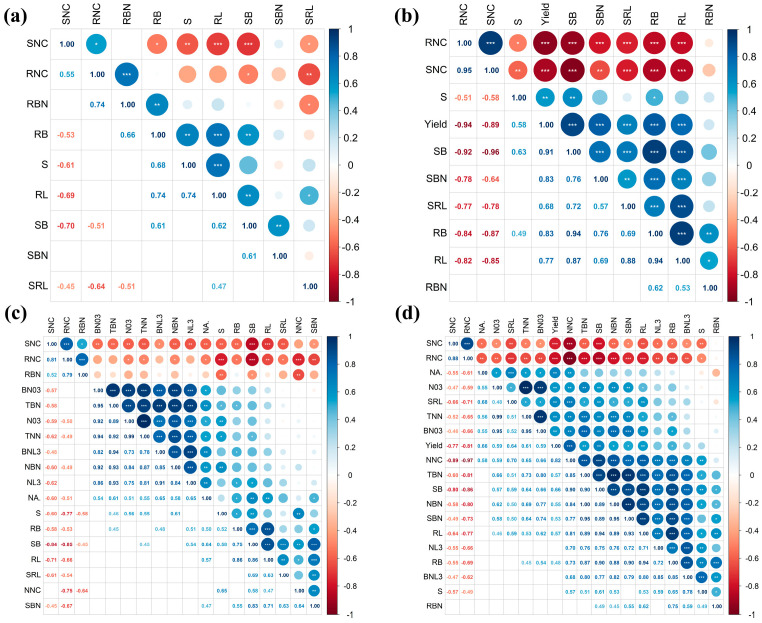
The Pearson correlation coefficients for plant indexes are as follows: (**a**) without rhizobia inoculation at the V9 stage, (**b**) without rhizobia inoculation at the R5 stage, (**c**) with rhizobia inoculation at the V9 stage, and (**d**) with rhizobia inoculation at the R5 stage. * indicates *p* < 0.05, ** indicates *p* < 0.01, *** indicates *p* < 0.001. S, sulfur concentration; SB, shoot biomass; RB, root biomass; RL, root length; SRL, specific root length; SNC, shoot N concentration; RNC, root N concentration; NNC, nodule N concentration; SBN, shoot biomass N accumulation; RBN, root biomass N accumulation; NBN, nodule biomass N accumulation; NA., nitrogenase activity; N03, number of nodules 0–3 mm in diameter; NL3, number of nodules larger than 3 mm in diameter; TNN, total number of nodules; BN03, biomass of nodules 0–3 mm in diameter; BNL3, biomass of nodules larger than 3 mm in diameter; TBN, total biomass of nodules.

Figure 8 illustrated strong correlations among various growth indicators of soybeans. Throughout different periods, shoot N concentration (SNC) and root N concentration (RNC) exhibited robust negative correlations with most indicators, regardless of rhizobia inoculation. In the absence of rhizobia inoculation, shoot biomass (SB) displayed the most prominent positive correlation with yield (*p* < 0.001), while root N concentration (RNC) demonstrated the most significant negative correlation with yield (*p* < 0.001) (Figure 8b). On the other hand, under rhizobia inoculation, root N concentration (RNC) maintained the strongest negative correlation with yield (*p* < 0.001), while nodule N concentration (NNC) displayed the most pronounced positive correlation with yield (*p* < 0.001) (Figure 8d). Interestingly, root N concentration (RNC) and nodule N concentration (NNC) exhibited a highly significant negative correlation (*p* < 0.001) during both the V9 and R5 periods (Figure 8c,d).

## 3. Discussion

### 3.1. The Impact of Sulfur on Soybean Growth

Soybeans, renowned for their affinity for sulfur, prove vulnerable to sulfur deficiency in the presence of ample quantities of the three primary elements—nitrogen, phosphorus, and potassium. As emphasized by Sexton et al. [33], excessive nitrogen input has the potential to disrupt the nitrogen-sulfur equilibrium, thereby inducing disorders in nitrogen metabolism, and ultimately impairing soybean growth and development. Almeida et al. [34] conducted experiments at 26 sites across 12 U.S. states and observed that the significant influence of sulfur fertilizers on yield, in contrast to the limited impact of nitrogen fertilizers, is primarily attributable to the high initial nitrogen content in the fields. Correspondingly, Brooks et al. [35] have noted that heightened nitrogen inputs prompt an increased demand for sulfur within soybeans. Miyatake et al. [24] discovered that deep application of controlled-release nitrogen fertilizers significantly increased soybean yield and nitrogen content, but these beneficial effects were hampered under sulfur-deficient conditions.

In alignment with earlier research [24], our investigation demonstrated that, irrespective of *Rhizobium* inoculation, the incorporation of sulfur led to substantial enhancements in aboveground nitrogen accumulation, aboveground biomass, and final soybean yields when compared to sulfur-deficient treatments. Sexton et al. [36] revealed that soybean leaves showed decreased sulfur and chlorophyll content, causing protein and photosynthetic pigment synthesis disruption and, consequently, reduced photosynthesis when soil sulfur content fell below 35 mg/kg. Boem et al. [37] found that sulfur deficiency primarily affects soybean yield by diminishing seed quantity. Another related study showed that applying sulfur fertilizer with finer particles to the soil increased albumin, globulin, prolamin, and glutelin protein contents [25]. Additionally, Rushovich and Weil [38] conducted experiments indicating that foliar sulfur fertilizer application significantly elevated methionine and cysteine levels in soybeans, while soil-applied sulfur fertilizer showed no significant effect. Thus, maintaining adequate nitrogen levels and strategically increasing sulfur application, considering both soil application and foliar spraying, can optimize the nitrogen-sulfur ratio in soybeans. This, in turn, stimulates nitrogen metabolism and protein synthesis, ultimately enhancing both soybean yield and quality.

As observed by Sugiura et al. [39], sulfur supplementation plays a pivotal role in enhancing soybean growth through the stimulation of organic acid secretion by soybean roots. These organic acids, in turn, enhance phosphorus solubility, reinforce phosphorus absorption, and elevate phosphorus utilization efficiency in soybeans, thereby promoting growth and increasing yields. Interestingly, sulfur fertilization does not invariably benefit soybean growth. Elevated soil sulfur levels can impede soybean potassium absorption and utilization, resulting in adverse consequences for soybean yields [40]. Chandra et al. [41] highlighted the impact of both sulfur deficiency and excess on soybean metabolism, yield, and seed quality, encompassing carbohydrates and storage proteins. In our study, sulfur supplementation did lead to increased soybean yields. However, as sulfur concentrations rose, soybean yields did not proportionally increase and, in fact, exhibited a slight decline. The research conducted by Moreira et al. [40] and Chandra et al. [41] offered a precise explanation of this phenomenon. Consequently, achieving higher soybean yields necessitates careful attention to the sulfur-to-potassium ratio in soybean cultivation.

### 3.2. The Influence of Rhizobia on Soybean Growth

*Rhizobium* and soybeans share a symbiotic relationship in which *Rhizobium* plays a pivotal role by converting atmospheric nitrogen into a plant-accessible form, thereby enhancing nitrogen nutrition for leguminous crops. As demonstrated by Zhou et al. [42], soybean leaves benefiting from rhizobial inoculation exhibit heightened photosynthetic rates, stomatal conductance, and maximum photosynthetic rates. These improvements lead to more efficient light energy utilization, ultimately promoting soybean growth and increasing yields. Empirical studies consistently reveal that soybean plants subjected to rhizobial inoculation exhibit increased nitrogen accumulation and higher dry matter compared to non-inoculated counterparts. Consequently, rhizobial inoculation emerges as a viable strategy to augment nitrogen nutrition in soybean plants [43]. Moreover, Albareda et al. [44] have highlighted that augmenting the number of rhizobia in the inoculant further intensifies nodulation, seed yield, and seed nitrogen content. However, our investigation uncovers a crucial caveat: the efficacy of rhizobial inoculation in enhancing nitrogen nutrition in soybean plants is contingent upon sulfur’s presence. Without sulfur supplementation, rhizobia appear unable to induce elevated nitrogen accumulation in either the aboveground or belowground components of soybeans.

Qin et al. [45] explored the influence of *Rhizobium* inoculation on soybean’s phosphorus acquisition, observing varying effects dependent on phosphorus sources. Their research revealed that *Rhizobium* inoculation enhanced soybean’s phosphorus acquisition, with superior results in calcium-phosphorus-dominated soils. Wang et al. [46] similarly demonstrated that rhizobial inoculation significantly improved nitrogen (N) and phosphorus (P) utilization by soybean under conditions of low N and/or low P. Notably, the growth-promoting benefits of rhizobial inoculation were less evident under high N and/or high P conditions. These findings align closely with the outcomes of Alam et al. [47], who reported that rhizobial inoculation markedly increased the number of nodules, nodule weight, shoot and root biomass, as well as enhancing nitrogen fixation enzyme activity and yield in soybean plants compared to non-inoculated soybean plants.

### 3.3. Sulfur and Rhizobia Combined Impact

Sulfur exerts a noticeable influence on soybean root nodule growth. Zhao et al.’s study [48] demonstrated that sulfur application significantly increases both the quantity and mass of soybean root nodules, aligning with our research findings. Indeed, sulfur application unequivocally enhances the number and mass of soybean root nodules compared to its absence. Furthermore, as sulfur concentrations rise, there is a noticeable shift in the proportion of large and small nodules, accompanied by a substantial increase in the dry weight of individual large nodules. Previous research has highlighted that soybeans with a higher number of large nodules exhibit heightened nitrogenase activity compared to those with smaller nodules. However, the excessive enlargement of nodules can potentially impede plant growth by encroaching upon the spatial territory of soybean roots, thus hindering soybean development [49]. In our study, it is plausible that at elevated sulfur concentrations, the significant enlargement of nodules has fostered a closer connection between nitrogenase activity and the abundance of small nodules.

Sulfur deficiency results in a reduction in root nodules and hampers symbiotic nitrogen fixation in soybeans [50], a phenomenon affirmed in our study. Even when inoculated with *Rhizobium*, a sulfur concentration of 0 mM failed to produce root nodules, significantly reducing nitrogen accumulation and soybean shoot biomass. Wooding et al. [51] reported that sulfur deficiency inhibited nitrogen fixation and nitrogen metabolism in nodulated soybeans, leading to decreased nitrogen content. In contrast, non-nodulated soybeans showed no impact on nitrogen metabolism and content under sulfur deficiency. This contrasts with our findings, possibly due to Wooding et al. [51] not observing the entire soybean reproductive period. Our study revealed that nitrogen accumulation in shoots and roots of non-nodulated soybeans remained unaffected by sulfur concentration during the early growth stages (V9). However, during the later growth stage (R5), sulfur deficiency significantly reduced nitrogen accumulation in the shoots of non-nodulated soybeans. The variation in sulfur-regulated nitrogen fixation in non-nodulated soybeans during different periods (V9 and R5) may stem from soybean’s lower nitrogen demand [52] before entering the reproductive stage. Once in the reproductive stage, soybeans need to synthesize more proteins for seed development [53], and sulfur deficiency inhibits protein synthesis and nitrogen metabolism [41].

### 3.4. Roots and Nodules in Soybean

Roots serve as the principal avenue for nutrient uptake in soybeans, with root nodules playing a pivotal role in enhancing nutrient acquisition. In our study, soybean plants bearing nodules demonstrated a notable yield increase, ranging from 30.94% to 119.48%, compared to nodule-free soybean plants in similar conditions. Consequently, *Rhizobium* inoculation and ensuring its persistence are critical factors for augmenting soybean yield. The survival of soybean rhizobia in soil is contingent upon various factors, including the specific strain, soil type [44], pH, soil organic matter, nutrients, salinity, agricultural practices, and temperature and moisture [54]. Bacterial microbiota play a significant role in shaping soybean rhizobium-host interactions and possess the capacity to influence the morphology and structure of root nodules. Research has indicated that distinct bacterial communities can impact nodule size, shape, and number, thereby influencing soybean’s nitrogen uptake and utilization efficiency [55]. Thus, the exploration of indigenous soybean symbiotic nitrogen-fixing bacteria, adapted to diverse environmental conditions, holds great promise for enhancing soybean yield and promoting sustainable agricultural development [56].

However, an intriguing observation emerges from Figure 2b,d during the R5 stage of soybean growth: in the sulfur-supplemented treatment, the root length and specific root length of non-rhizobia-inoculated plants surpass those of rhizobia-inoculated counterparts at the same sulfur level. This observation suggests that root nodules may have adverse effects on the soybean root system. Furthermore, Figure 8c,d illustrates a highly significant negative correlation (*p* < 0.001) between root nitrogen concentration (RNC) and nodule nitrogen concentration (NNC). This indicates that as nodule nitrogen uptake capacity intensifies, root nitrogen uptake capacity diminishes. Research by Ishikawa et al. [57] sheds light on this phenomenon, demonstrating that nitrate-induced inhibition of nitrogen metabolism in soybean root nodules actually enhances nitrogen metabolism in soybean roots. Consequently, root nodules play a pivotal role in facilitating enhanced nitrogen acquisition and heightened soybean yields. Nonetheless, the heightened activity of nodules might lead to reduced engagement of the soybean root system. Furthermore, in a study by Hardarson et al. [58], the correlation between nitrogen fixation by rhizobia and nodule placement in soybean was investigated. Their findings revealed that nodules situated in the lower sections of the root fixed more nitrogen than crown nodules. Therefore, meticulous attention to and regulation of nodule proportions and location on roots bear significant importance in advancing soybean nitrogen fixation and yield improvement.

## 4. Materials and Methods

### 4.1. Rhizobia Culture Experiment

In this experiment, we tested three rhizobia strains: BDYD1, BXYD3, and USDA110. BDYD1 and BXYD3 were isolated and purified from soybean plant nodules in Boluo and Yingde field sites in South China [59]. USDA110 originated from USA soybean nodules [60]. To identify suitable strains for inoculation, we prepared six sets of YMB (yeast mannitol broth) liquid medium, each with different sulfur concentrations (0, 0.8, 1.6, 3.2, 6.4, 15.0 mM). The YMB medium consisted of mannitol 10 g/L, MgSO_4_·7H_2_O 0.2 g/L, NaCl 0.1 g/L, yeast powder 3 g/L, K_2_HPO_4_ 0.25 g/L, KH_2_PO_4_ 0.25 g/L, and CaCO_3_ 3 g/L, and was added to a constant volume with ultrapure water. The sulfur concentration was adjusted using anhydrous sodium sulfate. The inoculated Erlenmeyer flasks were placed in a constant temperature shaker, and shaken at 180 r/min for cultivation. To serve as the control, a non-inoculated liquid medium was used, and we continuously measured the OD (optical density) value for 6 days using a spectrophotometer at a wavelength of 600 nm.

### 4.2. Soybean Hydroponic Experiment

The experiment took place in the greenhouse of the College of Resources and Environment, South China Agricultural University (23°09′31.70″ N, 113°21′45.85″ E). It was designed as a two-factor hydroponic study, involving two types of rhizobia inoculation methods and five sulfur concentrations. The rhizobia inoculation methods included (+R) rhizobia inoculation and (−R) no rhizobia inoculation, while the sulfur concentrations were 0, 0.15, 0.30, 0.60, and 1.20 mM. In total, there were 10 treatments, each replicated four times. The experimental design followed a completely randomized approach.

#### 4.2.1. Strain Preparation

The YMB medium was divided into Erlenmeyer flasks, which were then sealed with parafilm and sterilized in a sterilizing pot at 121 °C for 20 min. After inoculating the medium, the Erlenmeyer flasks were placed in a constant temperature shaker at 28 °C and shaken at 180 r/min for 4 days. To establish the baseline, the spectrophotometer was zero-adjusted using a liquid medium that had not been inoculated with bacteria, and the desired OD value was obtained when it reached approximately 1.0.

#### 4.2.2. Soybean Transplant

The test material used in this study was soybean (Yuechun 03-3), and the rhizobia strain employed for inoculation was USDA110. After completing soybean germination, seedlings with uniform growth were selected and soaked in a bacterial solution for approximately 1 h. Subsequently, they were transferred to a hydroponic box (37.5 cm × 27.5 cm × 14.5 cm) for the inoculation treatment, while seedlings that were not soaked in the bacterial solution served as the control group. Each hydroponic box accommodated 4 seedlings. The nutrient solution used in all hydroponic tanks comprised 8 L of improved Hoagland’s nutrient solution (pH = 5.8~6.0), formulated as follows: KNO_3_ 1.167 M, Ca(NO_3_)_2_·4H_2_O 0.667 M, NH_4_NO_3_ 0.750 M, CaCl_2_ 0.40 M, EDTA-FeNa 0.05 M, MnSO_4_·H_2_O 0.001879 M, ZnSO_4_·7H_2_O 0.001874 M, CuSO_4_·5H_2_O 0.000626 M, (NH_4_)_6_Mo_7_O_24_·4H_2_O 0.000202 M, NaB_4_O_7_·10H_2_O 0.003 M, KH_2_PO_4_ 0.625 M. Sulfur concentration was adjusted using K_2_SO_4_ and MgSO_4_·7H_2_O, and potassium and magnesium elements were supplemented using KCl and MgCl_2_. The pH of the nutrient solution was adjusted to 5.8~6.0 using 0.1 M dilute HCl and NaOH. Finally, a ventilation tube was connected, and the ventilation time was set to 15 min per hour during the day and every 15 min per 2 h at night.

#### 4.2.3. Plant Sampling

Sampling was carried out at two key growth stages; specifically, 24 days after transplanting (V9 stage) and 42 days after transplanting (R5 stage), with soybean grains harvested at full maturity. During sample collection, the plants were meticulously uprooted from the soil and segregated into their respective aboveground and underground components. The aboveground segments were enclosed within spacious envelopes and subjected to heat treatment at 105 °C for 30 min, followed by a drying process at 75 °C until a constant weight was achieved, thus yielding the dry weight index.

The underground portions underwent thorough washing with water, then excess moisture was gently removed using absorbent paper towels, and approximately 6–8 root nodules, encompassing both lateral and main roots, were excised, weighed, and promptly placed into sterilized penicillin vials to determine nitrogenase activity. These vials were immediately capped, sealed, and stored in an icebox. The remaining root nodules were meticulously sorted and counted, with differentiation based on their diameters, distinguishing those measuring >3 mm from those measuring ≤3 mm. The data collected included both the counts and respective diameters of these root nodules. Finally, the entire underground segment was sealed in airtight bags and preserved in the icebox for subsequent analysis.

The underground portions of the plants were initially subjected to scanning to ascertain root parameters using a root scanner. Subsequently, these underground components were placed in smaller envelopes and subjected to an oven-drying procedure at a temperature of 105 °C for a duration of 30 min. The samples were further dried at 75 °C until they reached a consistent weight, facilitating the computation of the dry weight index.

#### 4.2.4. Sample Determination

Root parameters: The root system was subsequently subjected to scanning using an Epson Expression 1600 Pro root scanner (Model EU-35, Japan) and analyzed using WinRhizo Reg2009 software to obtain the root parameters.

Nitrogenase activity: Nodule nitrogenase activity was assessed through the acetylene reduction method, commonly referred to as ARA (acetylene reduction assay [61]).

Plant digestion and determination of nitrogen content: Samples from both the aboveground and underground portions of the plants were ground and digested using a sulfuric acid-hydrogen peroxide mixture for the purpose of quantifying their nitrogen content, employing a flow analyzer. Additionally, the root nodules were subjected to drying to determine their dry weight indicators, and their nitrogen content was also assessed using a flow analyzer.

#### 4.2.5. Statistical Analyses

Principal component analysis (PCA) and Pearson correlation coefficient analysis were conducted using R 4.1.0 software (R Core Team, Vienna, Austria). The permute, lattice, vegan, dplyr, and ggplot2 R packages were employed for data processing and graphics to generate principal component analysis plots. Meanwhile, the corrplot R package was used to process and create graphs for Pearson’s correlation coefficient analysis. For other data analysis, SAS 10.0 (SAS Institute Inc., Cary, NC, USA) served as the statistical software. To assess differences among means, the least significant difference (LSD) test was applied at a 5% probability level. The results were visually represented using GraphPad Prism 9 (GraphPad Software Inc., San Diego, CA, USA).

## 5. Conclusions

Our research findings indicated that the application of sulfur and rhizobia inoculation significantly impacted the soybean yield during cultivation. The application of sulfur and rhizobia inoculation, in contrast to the absence of both, led to a significant yield increase, ranging from 868.14% to 1222.07%. USDA110 was closely related to sulfur and was greatly affected by sulfur concentration. In the absence of sulfur supplementation, soybean growth, nitrogen fixation, yield, root morphology-related parameters, and root nodule growth were markedly inhibited. Furthermore, sole rhizobia inoculation without sulfur supplementation did not improve the growth and yield of soybean. While increasing sulfur supply, there was no discernible upward trend in soybean production capacity. Conversely, excessive sulfur application could adversely affect soybean growth and inflate cultivation costs. It may also heighten soybean reliance on root nodules while diminishing the roots’ nutrient acquisition ability. Sulfur fertilization and rhizobia inoculation prove indispensable for promoting soybean growth and enhancing yield. In this study, 0.15–0.30 mM is the ideal sulfur concentration to be used in soybean cultivation. However, given the expense of soybean cultivation, sulfur application should not be excessive. Even a modest sulfur supply, while inhibiting the root system to some extent, facilitated soybeans in acquiring a sufficient number of small nodules, curbing the formation of large nodules. This, in turn, enhanced nitrogenase activity, stimulated nitrogen fixation, augmented dry matter accumulation in soybean shoots, and ultimately amplified soybean yield.

Our findings suggest that future research should prioritize the investigation of nutrient uptake and transport mechanisms between soybean roots and nodules. While our study highlights the potential role of sulfur in facilitating nutrient absorption through nodules rather than the root system, the precise mechanisms governing the interactions between these two components remain unclear. Additionally, exploring the responses of soybean roots and nodules to drought stress mediated by sulfur is essential, especially considering that our experiment was conducted under hydroponic conditions, and the effects of the root system’s extent on nutrient uptake may differ in field plantings. Moreover, it is crucial to determine whether the effects of sulfur under drought stress align with those observed in hydroponic setups. Lastly, we should also consider the long-term implications of sulfur application and rhizobial inoculation on soil health and sustainability. A comprehensive understanding of the enduring effects of elemental sulfur, rhizobia, and their secretions on soil microbial communities, soil structure, and nutrient cycling will contribute to a more comprehensive assessment of the impacts of sulfur application and rhizobial inoculation on agro-ecosystems.

## Figures and Tables

**Table 1:** The influence of different sulfur concentrations on the quantity and dry weight of root nodules at the V9 and R5 stages following rhizobia inoculation. The category “<3 mm” refers to nodules with a diameter ranging from 0 to 3 mm, while “>3 mm” indicates nodules with a diameter larger than 3 mm.

Stage	Sulfur Concentration (mM)	Number of Nodules (No.·Plant^−1^)			Dry Weight of Nodules (g·Plant^−1^)		
		<3 mm	>3 mm	Total Number	<3 mm	>3 mm	Total Weight
V9	0	0 ± 0.0 b ^1^	0 ± 0.0 a	0 ± 0.0 b	0 ± 0.0000 a	0 ± 0.0000 a	0 ± 0.0000 a
	0.15	6 ± 1.2 ab	3 ± 0.3 a	9 ± 1.0 ab	0.0062 ± 0.0012 a	0.0049 ± 0.0004 a	0.0112 ± 0.0010 a
	0.30	11 ± 5.9 ab	3 ± 2.0 a	15 ± 6.1 ab	0.0052 ± 0.0018 a	0.0064 ± 0.0037 a	0.0116 ± 0.0047 a
	0.60	21 ± 10.6 ab	5 ± 1.6 a	26 ± 12.1 ab	0.0195 ± 0.0109 a	0.0134 ± 0.0047 a	0.0329 ± 0.0155 a
	1.20	26 ± 11.6 a	3 ± 2.3 a	30 ± 13.5 a	0.0161 ± 0.0087 a	0.0140 ± 0.0124 a	0.0301 ± 0.0204 a
R5	0	0 ± 0.0 c	0 ± 0.0 b	0 ± 0.0 c	0 ± 0.0000 b	0 ± 0.0000 c	0 ± 0.0000 c
	0.15	192 ± 52.3 ab	19 ± 8.2 a	211 ± 57.5 ab	0.5248 ± 0.1581 a	0.1925 ± 0.0685 bc	0.7344 ± 0.1966 b
	0.30	226 ± 59.2 a	18 ± 3.2 a	244 ± 57.6 a	0.5721 ± 0.1272 a	0.2577 ± 0.0356 b	0.8531 ± 0.1264 ab
	0.60	156 ± 18.0 ab	26 ± 1.4 a	182 ± 17.5 ab	0.5647 ± 0.0456 a	0.5857 ± 0.0662 a	1.1919 ± 0.0663 a
	1.20	75 ± 24.2 bc	28 ± 7.1 a	103 ± 17.5 bc	0.2255 ± 0.0408 b	0.5796 ± 0.1147 a	0.8695 ± 0.0903 ab

^1^ At the same stage, mean ± SE sharing a common letter within a column do not differ significantly at *p* < 0.05.

## Data Availability

All data reported here are available from the authors upon request.
